# Cardiovascular proteomics in the era of big data: experimental and computational advances

**DOI:** 10.1186/s12014-016-9124-y

**Published:** 2016-12-05

**Authors:** Maggie P. Y. Lam, Edward Lau, Dominic C. M. Ng, Ding Wang, Peipei Ping

**Affiliations:** 1 NIH BD2K Center of Excellence at UCLA; Department of Physiology, University of California at Los Angeles, 675 Charles E. Young Drive, Los Angeles, CA 90095 USA; 2 Department of Medicine, University of California at Los Angeles, 675 Charles E. Young Drive, Los Angeles, CA 90095 USA; 3 Department of Bioinformatics, University of California at Los Angeles, 675 Charles E. Young Drive, Los Angeles, CA 90095 USA

**Keywords:** Cardiovascular medicine, Clinical proteomics, Shotgun proteomics, Mass spectrometry

## Abstract

Proteomics plays an increasingly important role in our quest to understand cardiovascular biology. Fueled by analytical and computational advances in the past decade, proteomics applications can now go beyond merely inventorying protein species, and address sophisticated questions on cardiac physiology. The advent of massive mass spectrometry datasets has in turn led to increasing intersection between proteomics and big data science. Here we review new frontiers in technological developments and their applications to cardiovascular medicine. The impact of big data science on cardiovascular proteomics investigations and translation to medicine is highlighted.

## Background

The heart is in many ways an exceptional organ. Proteins at the sarcolemma, sarcomere, mitochondrion, and other cardiac organelles must orchestrate vital functions seamlessly on a beat-by-beat basis, while dynamically adjusting energetic and contractile outputs to environmental cues within seconds. Heart diseases including cardiac hypertrophy and failure are characterized by complex remodeling of various protein signaling networks and subcellular components, which often involve a multitude of collaborating proteins. Therefore, understanding how multiple protein species interact to carry out higher physiological phenotypes and regulation has been an important objective of cardiovascular research. The power of proteomics to simultaneously provide information on the panoply of expressed proteins has made it uniquely suitable for resolving complex signaling conundrums and revealing disease mechanisms in the heart.

Advances in genome sequencing are often celebrated to have outpaced even the vaunted Moore’s law of computing power [1]. Lesser known but equally impressive is the parallel surge in the capacity of mass spectrometry-based proteomics last decade. To wit, when the first draft of the human genome was published in 2001, a state-of-the-art two-dimensional electrophoresis technique could identify ~200 proteins in 3 days. Fast-forward to today, a modern mass spectrometer can generate over a million spectra per day and quantify ~4000 proteins in the course of 1 h [2]. This quantum leap is attributable to parallel advances in three areas: (1) analytical chemistry in sample processing and liquid chromatography tandem mass spectrometry (LC–MS/MS) instrumentation; (2) bioinformatics and computational tools in high-throughput data processing and analysis; and (3) completeness and accuracy of sequence and annotation databases. Riding on growing experimental capacity, there have been continued improvements to the experimental coverage of proteome analysis, the interpretability of data, the reliability of results, and the diversity of protein parameters that may be interrogated. New applications and experimental designs not possible a few years ago are now being exploited to explore new regulatory modalities in cardiac physiology.

**Fig. 1:**
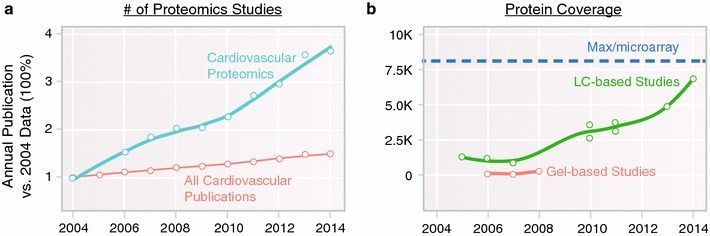
Trends in cardiovascular proteomics. Both (**a**) the volume of proteomics studies, and (**b**) the size of proteomics dataset have skyrocketed in the last decade. **a** The number of cardiovascular proteomics studies has increased approximately 400 % from 2004 to 2014, far outpacing the natural growth of the cardiovascular field, indicating increasingly common adoption of the technologies. **b** The protein coverage of proteomics experiments in the same time period has experienced considerable growth also, quantified as the numbers of identifiable cardiac proteins in an experiment. The maximum number of cardiac proteins (*dashed lines*) is based on estimated significantly expressed loci in the mouse heart and does not take into account proteoforms such as resulting from alternative splicing. This increase is driven by parallel advances in hardware instrumentation and computational technology. Coinciding with the notion of “complete proteomics”, proteomics studies can now interrogate more proteins of interest such as chromatin remodeling factors and transcription factors that express at low copy numbers. Effective means to analyze big proteomics dataset are becoming a new frontier of growth in cardiovascular proteomics

Cardiovascular proteomics has grown rapidly in the intervening period, with >400 studies now being published yearly (Fig. 1) [3]. To put into context, we recall two landmark reviews of cardiovascular proteomics, in 2001 [4] and 2006 [5], which noted that although many enabling technologies were emerging, cardiovascular proteomics remained a field ‘on the threshold’ of future applications. Fast forward to the present and it is clear that proteomics has had a transformative impact on cardiovascular sciences, as recounted in recent review articles. We attempt to complement these reviews here with a concise overview on the lockstep improvements in the analytical (separation sciences and mass spectrometry) and computational (data science and algorithms) advances of the past 5 years that enabled landmark studies, as well as ongoing developments driving the next stage of applications.

## Experimental and analytical advances

### Improvements in analytical methods

An early hurdle that bedeviled cardiovascular proteomics was the limitation in the sensitivity and dynamic range of protein detection, which skewed results towards few high-abundance proteins (e.g., contractile proteins) and masked low-abundance species. This is due to the complexity of proteomes. The mammalian heart is known to express at least ~8000 genes at significant levels [6], and at least 8325 human proteins have been referenced in the ~1.4 million cardiac-related publications on PubMed [7]. Each human gene can encode multiple proteoforms, e.g., due to the average ~4 alternative splicing isoforms per human gene plus many more post-transcriptional and post-translational editing processes, resulting in at least ~10^6^ proteolytic peptides per sample. With the addition of post-translational modifications (PTMs)—e.g., the four histone proteins alone have identified PTMs on at least 105 different residues in myriad combinations [8]—the total proteome complexity is likely orders of magnitude more complex still.

In the past decade, great strides have been made to improve proteome coverage, from how protein samples are extracted to mass spectrometry instrumentation. To perform proteomics analysis, it follows that the proteins must be effectively extracted and released from biological samples. This is typically achieved via mechanical homogenization or chemical surfactants. Protein solubilization techniques in early proteomics protocols were at times ineffectual in extracting hydrophobic or membrane proteins, which often aggregated out of the sample and led to their non-detection. Development in this area in recent years have led to commercially available, mass spectrometry compatible surfactants [9], size exclusion filter-mediated buffer exchange techniques [10], as well as empirically refined experimental protocols that are optimized for the analysis of various cardiac subproteomes [11]; all of which serve to expand the portion of the proteome that is open to mass spectrometry exploration. The incompleteness of proteolytic digestion was once found to be a limiting factor of the maximal peptide coverage of the experiment and contribute to batch-to-batch variations. The use of optimized proteolysis protocols including double lys-C/trypsin proteolysis is gaining traction [1].

**Fig. 2:**
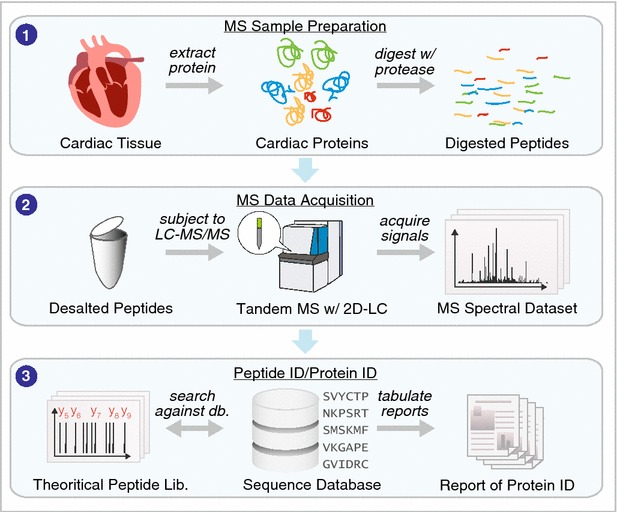
Analytical and computational overview in protein identification. *1* Cardiac samples are processed to extract the proteomes or subproteomes of interest, which may then be proteolyzed to obtain peptide digests. *2* The resulting peptides are desalted and subjected to LC–MS/MS analysis to acquire MS1 and MS2 spectra. *3* The peptide sequences that are present in the MS dataset can be identified using a database search approach, which uses a sequence database (e.g., UniProt) to generate theoretical peptide sequence and predict their fragmentation patterns in silico, then automatically find the best-match theoretical spectra to the experimental spectra for protein identification. Alternatively, the proteins can be identified using a spectral library search. The resulting protein datasets can be further analyzed to extract other biomedically meaningful information (see Fig. 4)

Advances in separation sciences have had a particularly tremendous impact on reducing the complexity of peptides prior to mass spectrometry signal acquisition. High-performance shotgun proteomics using mass spectrometry has supplanted two-dimensional (2D) electrophoresis to become the de facto standard for large-scale analysis of cardiac proteins (see general workflow of shotgun proteomics in Fig. 2). Whereas the now-dethroned 2D electrophoresis was limited to detecting a few hundred proteins, contemporary LC–MS experiments can resolve peptides from >10,000 proteins to allow their identification and quantification. Since 2001, separation science has led in a relentless pursuit to increase protein coverage [12, 13], with the success of strong cation exchange-reversed phase based MudPIT approaches followed successively by other 2D-LC approaches including reversed-phase-reversed-phase separation [14] as well as very-long separation columns with high peak capacity, nano-scale microfluidic devices driven by ultra-high pressure LC systems [15] and capillary electrophoresis separation (see [16, 17] for reviews on separation science developments). By separating the peptide samples into smaller subset based on their chemistry, a simpler mixture of peptides is introduced into the mass spectrometer in any given time, which decreases ion competition and increases sensitivity.

State-of-the-art instruments including hybrid Orbitraps and time-of-flight instruments achieve high performance by virtue of their high scan speed (allowing more peptides to be analyzed in the same analysis), sensitivity (allowing minute amounts of samples to be analyzed), and mass resolution (increasing power to differentiate similar peptide species). Recent proteome profiling experiments of the mammalian heart using the latest and greatest LC–MS combinations routinely achieve 5000 or more proteins identified in an experiment (Fig. 1b). In a recent survey we quantified the relative abundance of 8064 proteins in the mouse heart, covering more than 10 major organelles and 201 major cellular pathways [18]. As little as micrograms of proteins are sufficient for shotgun proteomics analysis. This amount may come from milligrams or less of cardiac biopsies, or ~10^5^ cultured cardiac cells, opening the door of proteome analysis to more experimental and clinical designs where sample amounts may be limiting.

Taken together, these advances have helped solve a principal challenge to proteomics applications, namely how to successfully detect the maximal number of peptides inside the overwhelmingly complex mixture that is the cardiac proteome. Although each technological development is incremental, over time they accrued into a qualitative transformation on the power and utility of proteomics, when proteins of biomedical interest gradually became measurable and discoverable in large-scale experiments. Catalogs of so-called “complete proteomes” (i.e., here narrowly defined as the detection of one protein product of every expressed locus in the genome) have now been described for many human tissues and organs, including the heart [19, 20]. Therefore, although earlier cardiovascular proteomics studies were best equipped to discover changes in structural, contractile, or metabolic housekeeping proteins, contemporary studies can now easily interrogate regulatory proteins including membrane receptors, kinases, ubiquitin ligases, and chromatin remodeling factors, whereas the analyses of yet scarcer species such as transcription factors and cytokines are now on the cusp of routine applications.

Early applications were also plagued by the variability of quantification results, which limited power to discover significant changes between disease model and control samples. A major source of variability in proteomics quantification originated from the variable detectability of peptides with different amino acid compositions. Two equimolar peptide sequences can show rather different intensities in MS signals. Accurate prediction of peptide intensity based on sequence information remains an unsolved issue in computational proteomics due to the large number of combinatorial variables that contribute to signal behaviors. Several methods have been developed to normalize peptide intensity and achieve accurate quantification. Targeted MS methods, such as Multiple Reaction Monitoring (MRM) [21], allow users to program the mass spectrometer to scan for only targeted peptide ions for quantification. An advantage of targeted MS is the gain in reproducibility and sensitivity, which can avail the detection of low-abundance proteins at their native concentration. Targeted assays have been successfully developed such that very low amount of proteins in the sample can be quantified, as in the case of troponin I [22]. Isotope labeling methods including SILAC and Super-SILAC [23, 24] can also reduce variability in relative quantification by ensuring peptides from multiple samples are compared in identical experimental conditions, but require additional labeling steps.

**Fig. 3:**
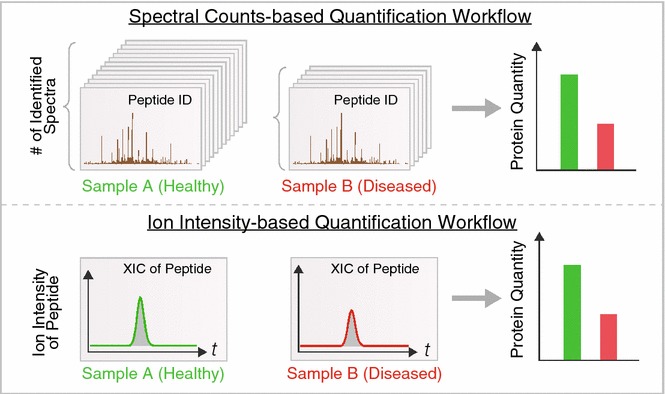
Common label-free quantification in proteomics studies. Two common label-free quantification approaches in use are based on spectral count (*top*) and ion intensity (*bottom*). *Top*: spectral counting methods leverage the fact that in stochastic shotgun profiling, the frequency of a protein being sampled by the instrument scales with its relative abundance in the sample. The numbers of spectra matched to an identical protein in healthy (*green*) versus diseased (*red*) samples can therefore be compared if appropriate normalization and bioinformatics workflows are implemented. *Bottom*: ion intensity methods integrate the total signal intensity of peptide ion signals in the mass spectrometer to infer protein quantity. Software is now available to automatically identify and quantify peptide ion signals from mass spectra

With advances in data acquisition methods, non-targeted label-free techniques can also reliably deduce accurate protein intensity from shotgun experiments directly through bioinformatics analysis. Label-free quantification is analogous to deducing transcript abundance from read counts in next-generation sequencing. Existing approaches largely fall into two categories (Fig. 3). Spectral counting exploits the bias of shotgun proteomics towards abundant proteins, and calculates protein quantity from the stochastic sampling frequency of peptide ions, i.e., the higher the protein abundance, the more of its MS spectra are likely to be identified. A major advantage of spectral counting is that it quantifies directly from the identification output and thus is compatible with most workflows. Spectral counting algorithms tally the number of redundant spectra for each identifiable peptide, then sum the numbers of spectra for all peptides assigned to a protein. On the other hand, ion intensity approaches integrate the intensity mass-specific ion signals over time in the chromatographic space. This utilizes a feature detection step in data analysis to read raw MS files and integrate the corresponding areas-under-curve of each peptide ion over time. Both labeled and label-free methods provide a useful guide to differential protein expression, and can now be used to discover candidate disease protein that can then be validated by further studies.

### Improvements in software tools

The massive amount of MS data generated in proteomics experiments requires computational aid for effective data processing and analysis. A growing number of open-access computational tools concerning all steps of proteomics data analysis are now freely available to users, a subset of which are listed in Table 1.

**Table 1:** Selected open access software tools in proteomics

Open access tools	Language/framework	License	Publication	Website
*Database search engine (untargeted proteomics)*				
Comet*^,†^	C++	Apache 2.0	[93]	[94]
MS-GF+*^,†^	Java	Custom/Academic	[31]	[95]
MSAmanda^†^	C#/Mono	Custom/Academic	[96]	[97]
ProLuCID*^,†^	Java	Custom/Academic	[26]	[98]
X!Tandem*^,†^	C++	OSI Artistic	[99]	[100]
*Targeted proteomics and/or data-independent acquisition*				
Skyline*	C#	Apache 2.0	[101]	[102]
OpenSWATH*^,†^	C++	BSD 3-Clause	[103]	[104]
*Protein inference and/or search post-processing*				
Percolator*^,†^	C++	Apache 2.0	[34]	[105]
ProteinProphet^*,†^	C++	GNU LGPLv2	[106]	[107]
ProteinInferencer^†^	Java	Custom/Academic	[35]	[98]
*Protein quantification*				
MaxQuant	.NET	Custom/Academic	[108]	[109]
Census^†^	Java	Custom/Academic	[110]	[98]
PLGEM^*,†^	R	GNU GPLv2	[111]	[112]
QPROT^*,†^	C	GNU GPLv3	[37]	[113]
*Pipelines and toolkits*				
Perseus	.NET	Custom/Academic	[114]	[115]
Crux*	C++	Apache 2.0	[116]	[105]
OpenMS*	C++	BSD 3-Clause	[117]	[118]
TPP*	C++	GNU LGPLv2	[119]	[120]
*Data access and reuse*				
PeptideShaker*	Java	Apache 2.0	[121]	[122]
PRIDE inspector*	Java	Apache 2.0	[123]	[124]

Proteomics software tools that provide open access to users. Many of these tools are also open source which potentially allows users to participate in the continual development of the tools

* Available open-source source code repository at the time of writing

^†^Platform-independent (Windows, Linux, Mac)

A major computational task in shotgun proteomics is to efficiently interpret the mass and intensity information within mass spectral data to identify proteins. The computational task can be formulated thus: given a particular tandem mass spectrum, identify the peptide sequences most likely to have given rise to the set of observed parent molecular mass and fragment ion patterns in a reasonable time frame. A general solution to this problem is “database search”, which involves generating theoretical spectra based on in silico fragmentation of peptide sequences contained in a protein database, and then systematically comparing the experimental MS spectra against the theoretical spectra to find the best peptide-spectrum matches. The SEQUEST algorithm was the first proposed to solve this peptide spectrum matching problem in 1994 [25] and its variants (e.g., Comet, ProLuCID [26]) remain among the most widely utilized algorithms to-date for peptide identification. SEQUEST-style algorithms score peptide-spectrum matches in two steps, with the first step calculating a rough preliminary score which empirically restricts the number of sequences being analyzed, and the second step deriving a cross-correlation score to select the best peptide-spectrum match among the candidates. Recent descendants of the SEQUEST algorithms have focused on optimizing its searching speed as well as improving the statistical rigor of candidate sequence scoring, with some programs reporting ~30 % more peptides/proteins identifiable from identical MS datasets and better definition of true-/false-positive identifications [26–29]. Other search engines also exist that are commonly in use, including X!Tandem, which calculates the dot product between experimental and theoretical spectra, then derives the expectation value of the score being achieved in a random sequence match; MaxQuant/Andromeda, which considers fragment ion intensities and utilizes a probabilistic model for fragment observations [30], MS-GF+ [31], and others. Methods have also been developed to combine the unique strengths and biases of multiple search engines to improve total protein identifications [32].

Means to distinguish true and false positives are critical to all large-scale approaches. The “two-peptide rule” was once commonly adopted to decrease false positives at the protein level by requiring each protein to be identified by at least two independent peptides. However, this rather conservative rule could inflate false negatives, as some short or protease-incompatible proteins may only produce maximally one identifiable peptide. More recent conventions involve foregoing the two-peptide rule and instead estimating the false discovery rate (FDR) of identification through statistical models, often with the aid of decoy databases. The use of decoy databases/sequences (reversed or scrambled peptide sequences), allows a quick estimation of the number of false positive proteins, by assuming identical distribution in protein identification scores for false positive hits and the decoy hits. A maximum acceptable FDR can then be specified (conventionally 1–5 %) to determine which protein identifications are accepted in the final result. To explicitly reveal the posterior probability of any particular identification being correct (also called the local FDR), a mixture model has been used that assumes that the peptide identification result is a mixture of correct and incorrect peptides with two distinct Poisson distributions of identification scores [33]. Auxiliary determinants including the presence of other identified peptides from the same proteins can also be applied to infer overall likelihood of protein assignment [33]. Machine learning algorithms (e.g., Percolator) have been demonstrated to build classifiers that automatically distribute peptide spectrum matches into true and false positives [34]. New inference approaches have also been demonstrated that consider peptide and protein level information together to improve the confidence of identification [35].

**Fig. 4:**
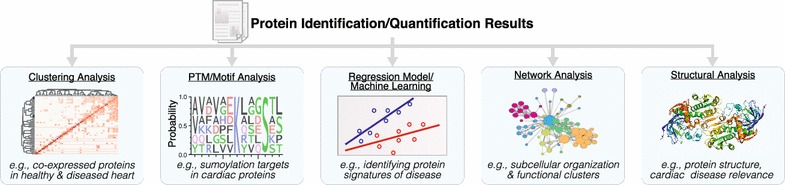
Proteomics data mining and functional annotations. Common computation approaches to extract information from massive proteomics datasets include (1) unsupervised cluster analysis, class discovery and visualization; (2) motif analysis and annotation term enrichment; (3) statistical learning methods for disease signature extraction; (4) network analysis; and (5) annotations with other functional information including protein motifs and cardiac disease relevance

With the increase in data size and multiplexity (number of sample compared) in proteomics experiments, statistical approaches to analyze data have also evolved to tackle high-dimensionality data. Whereas early studies utilized mostly confirmatory statistics, modern proteomics datasets typically contain thousands of features (e.g., protein expression) over a handful of observations, hence simply testing whether each protein is significantly altered across experimental conditions can result in under-analysis and failure to distinguish latent structures across multiple dimensions, e.g., whether there exists a subproteome of co-regulated proteins across multiple treatment categories. To gain biological insights, quantitative proteomics datasets are now routinely mined using statistical learning strategies that comprise feature selection (e.g., penalized regression methods), dimensionality reduction (e.g., principal component analysis), and both supervised and unsupervised learning (e.g., support vector machine and hierarchical models) to discern significant protein signatures, disease-implicated pathways, or interconnected co-expression networks (Fig. 4).

Improvements to computational methods that allow more robust results from label-free quantification are an area of active research. For example, recent works (e.g., QProt) have attempted to resolve the respective quantities of multiple proteins that share common peptide sequences in spectral counting, either using weighted average methods or more statistically motivated models [36, 37]. In ion intensity approaches, chromatographic features that correspond to peptide signals over mass- and retention time-dimensions are identified using image analysis or signal processing algorithms. Because LC gradients are seldom perfectly reproducible, nonlinear distortions in retention time may occur. To ensure identical ions are compared between experiments, automatic chromatographic alignment and clustering methods are used. Some software can identify small chromatographic features based on accurate mass/retention time alone, such that some peptides may be quantified even in experiments where they were not explicitly identified. These processes tend to become computationally expensive for large experimental files [38], thus faster solutions are continuously developed.

With the proliferation of inter-compatible tools, an ongoing trend is to daisy-chain individual tools into user-friendly pipelines that provide complete solutions to a set of related data analysis problems. An ideal proteomics pipeline may combine identification, quantification, and validation tools in a modular organization accessible from a single location. Computation may be performed on the cloud to avoid the need to repeatedly copy, transfer, and store large files. Researchers can carry out computational tasks remotely from the browser on any computer system, obviating the need for redundant infrastructure investments. Currently, the Trans-Proteomics Pipeline [39] and the Integrated Proteomics Pipeline [40] are two example “end-to-end” pipelines that connect raw MS proteomics data to analysis output, whereas comprehensive, open-access pipelines have also been demonstrated in other omics fields, including Galaxy for genomics/transcriptomics [41] and MetaboAnalyst for metabolomics [42]. In parallel, tools are also being federated into interoperable networks through open frameworks. A modular and open-source software development paradigm, where individual software functionalities can interoperate via common interfaces and standards, helps ensure that new software can dovetail with existing ones with ease, and that software development may continue following inactivity from the original research team. Examples of such frameworks include the GalaxyP proteomics extension [43], and the proteomics packages within the R/BioConductor framework [44].

### Improvements in annotation resources

Not unlike other omics approaches, the success of proteomics experiments relies heavily on having adequate and up-to-date resources to analyze large-scale data. To wit, for protein database search to succeed, it follows that the protein being identified must first be documented on a sequence database. Fortunately, there have been tremendous advances on the completeness (number of true positive sequences recorded) and precision (removal of redundancy or artifacts) of sequence databases such as UniProt and RefSeq. Some databases are manually curated to contain precise information, whilst others strive to be more comprehensive; but many now list precise “complete proteomes” for commonly studied laboratory species. Databases for human and popular laboratory model organisms have seen particular progress in the completeness of annotation in recent years, such that proteomics studies can now be performed similarly well in mice, drosophila, rats, and other organisms to interrogate cardiac physiology. On the horizon, one can foresee an influx of sequence information on protein polymorphism and alternative splicing isoforms. Although current databases primarily originate from genomic translation or cDNA library of specific cell types or genetic backgrounds, “proteogenomics” efforts are underway to translate additional sequences for proteomics studies, which will expand the scope and precision of protein identification. For non-model organisms, alternative search methods have been devised, such as against a custom database generated by manual six-frame translation of genomic sequences.

Data interpretability problems arise when the complex results comprising changes of many proteins could not be easily digested and summarized in terms that are relatable and of value to biomedical and clinical researchers. The biological significance of the implicated protein targets may be interpreted and connected to the growing corpus of biomedical knowledge through functional annotations. Commonly used annotation resources include Gene Ontology for biological functions [45], Reactome or KEGG for curated biochemical and signaling pathways [46], PFAM for protein motifs and homology [47], PhosphoSitePlus for PTMs [48], and so on.

To map molecular data to curated annotations, a class discovery approach is commonly utilized, which looks for annotated properties that are preferentially shared amongst a subset of proteins with interesting quantitative features over the proteome-wide background, e.g., through sequence motif analysis or annotation term enrichment. The principle behind such analyses is to infer biologically relevant processes based on significant overlaps between data features and the data annotation classes. Computational and statistical approaches are used to determine whether particular annotations are over-represented in a particular subset results than would be expected by chance. This allows both bias-free discovery and specific questions to be asked of a dataset, e.g., whether the down-regulated proteins in a heart failure patient are preferentially involved in fatty acid metabolism, etc. Enrichment analyses can be easily carried out using online tools that perform binomial or hypergeometric tests on the over-representation of functional annotations, including NCBI DAVID, WebGestalt, and Pantherdb [49–51]. For users conversant in statistical programming and data analysis environment, open-access packages dedicated to proteomics data operation have been made available in languages such as R and Python, including the RforProteomics package [52] for MS data visualization on the R/Bioconductor repository, and the Python Pyteomics library [53] for parsing and processing MS data. These packages allow users to connect upstream MS analysis to downstream functional annotation services that are commonly employed.

We note that many proteomics functional analysis strategies were originally developed for microarray datasets. Although the analytical goals between proteomics and microarray experiments often overlap, i.e., identify functional associations from numerical molecular expression data, several methodological differences merit considerations. The stochastic nature of shotgun proteomics can lead to missing values and high variability in the data for low-abundance proteins. To address this, several proteomics-focused enrichment analysis tools have recently been developed to address specific features of proteomics datasets, e.g., using weighted sampling, which should account for relative abundance and variability of observations and further improve quantification performance. Monte Carlo sampling approaches have also been used to counter the bias of annotations on high abundance proteins when calculating enrichment significance [54].

As in the case for protein identification, the success of functional analysis is contingent upon the completeness and accuracy of annotations in knowledgebases. Commonly used knowledgebases such as Uniprot and Reactome have steadily improved in size and richness of functional information. Nevertheless, it sometimes remains the case that some classes of annotations are more complete, either because they are easily computable from sequence information (e.g., do these phosphoproteins with increased phosphorylation in heart failure share putative kinase domains?) or can be derived from popular experimental designs (e.g., whether a particular cardiac protein localizes to the mitochondrion?), and as a result are more likely to turn up in functional enrichment analyses. On the other hand, annotations on higher-level pathophysiology and tissue-specific regulations are more challenging, because a majority of such information is hidden in unstructured free text in the literature. With >2 million cardiovascular related articles alone on PubMed [55], however, the volume of literature data being published dwarfs the capacity of human expert biocurations to translate them. Hence in recent years many approaches are being pursued to improve biocuration, including crowdsourcing initiatives to leverage public contributors through web-based micro-tasks, as well as text-mining algorithms that comb through the literature and automatically convert free texts into computable formats. Domain-specific knowledgebases (including organelle specific databases [56], and cardiovascular disease specific knowledgebases [57] have also been developed to provide richer cardiovascular contexts in data interpretation.

## Examples and frontiers in cardiovascular applications

The aforementioned analytical and computational advances have enabled novel and noteworthy applications in cardiovascular proteomics. An exciting trend is to venture beyond simply inventorying which proteins are present in the heart or the blood, and into quantifying their dynamic and spatiotemporal properties. Protein complexity necessitates that many parameters are needed to sufficiently describe the overall proteome in a particular physiological state. New methodologies continually arise that enable new insights into protein–protein interactions [58], protein homeostasis [59], and spatial distributions [60]. Many molecular parameters are now known to be directly involved in disease pathogenesis thanks to proteomics studies, including the PTMs of proteins, their spatiotemporal distributions, and interacting partners.

### Quantifying diverse post-translational modifications

With increased experimental power to detect peptides, rare and hard-to-detect peptides are increasingly analyzable, including many modified by PTMs. Because translational modifications are attached to proteins following synthesis, their chemical identity, position, and fractional quantity cannot be easily predicted from transcripts, necessitating proteomics studies. PTMs have been the focus of proteomics studies for over a decade and these efforts have increasingly begun to bear fruit in various biomedical investigations. In a recent notable example, Lee et al. [61] used a global approach was used to discern the roles of phosphodiesterase (PDE) in cyclic guanosine monophosphate (cGMP) degradation in cardiac signaling. Although once assumed to be a common pathway acting through a single secondary messenger, the subcomponents are modulated by two different enzymes PDE5A and PDE9A at different cellular locations. A global, high-throughput phosphoproteomics profiling approach was used to differentiate the downstream signaling targets of the two pathways, allowing their precise regulations to be elucidated and subclassified. Therapeutic decision and precision medicine may be informed by targeting PDE9A versus PDE5A with different pharmacological compounds.

Evidence suggests that the current investigations into PTMs have barely scratched the surface of their complexity. Over 380,000 PTM events are documented on the PhosphoSitePlus database [48], including acetylation, di-methylation, mono-methylation, O-GlcNAcylation, phosphorylation, sumoylation and ubiquitiylation, etc. on a variety of proteins. But many additional, unknown modifications likely lurk in acquired spectra that await identification, which are the subject of ongoing developments such as using cascade search, open search, or spectral clustering approaches [62, 63]. In addition to classical studies of phosphorylation and ubiquitination, improved methods to isolate and identify PTMs have fueled investigations into many different kinds of modifications including glycosylation, acetylation, sumoylation, and oxidative modifications that are now known to play critical and indispensable roles in the regulations of core aspects of cardiac physiology. Publications from multiple groups have led to increasing appreciation of the fine regulations of oxidative cysteine modifications (including disulfide bridge, S-nitrosylation, and S-glutathionylation) in cardiac redox regulation [64]. Examples include the discovery of S-nitrosylation at TRIM72 in regulating ischemic injury [65], and the unexpected “moonlighting” of GAPDH in the mitochondria to confer S-nitrosylation in the heart [66]. An increasing number of other examined modifications are likewise now implicated in important cardiac processes, at a rate that far exceeds that which would be attainable in traditional single-target, hypothesis-driven investigations. These include for instance the role of acetylation in the context of mitochondrial metabolism [67, 68]; sumoylation in the context of heart failure [69]; O-GlcNAcylation in the context of diabetic hearts [70], and lysine succinylation in the context of ischemic injury [71]. These studies represent a broadening of our observable universe, and are driven by both advances in specific purification or labeling strategies, as well as a general increase in MS instrumentation and data analysis maturity that together propel the experimental scope, scale and reproducibility past the threshold of informativeness.

### Tracing protein spatiotemporal distributions

The function and functionality of a protein are modulated to a great extent by the spatial milieu in which it is situated, which in turn dictates the substrates and interacting partners with which it comes across. Ongoing studies into the spatial distributions of cardiac proteins are inventorying the protein compositions in different cardiac organelles, with particular successes in cardiac mitochondria and nucleus proteomes, as elucidated via targeted enrichment of specific organelles. More recent studies in other organs are suggesting the possibility that proteins are actively and constantly translocalizing between organelles in response to cues, which can be measured by combining differential centrifugation, isotope labeling, and machine learning techniques. Recent advances in data analysis have allowed such differential centrifugation techniques to be used for pan-organellar mapping. Instead of enriching only for a pure sample of a particular organelle type, a centrifugation gradient here is matched to a supervised classification algorithm to classify proteins based on their sedimentation behaviors. The average intracellular position of many proteins can therefore be discovered by their grouping with known organellar markers. These approaches can be readily adopted to understand dynamic protein translocalization from one organelle to another, using new analytical frameworks that can quantify protein translocation in differential centrifugation experiments [60, 72]. Through proteomics studies, it is also demonstrated that many proteins important in the heart can have multiple localizational isoforms that carry out different functions [73].

Secondly, the synthesis rates of proteins have also proven important to tracing the progression of cardiac hypertrophy preceding heart failure. Interests in classical physiology to understand skeletal and cardiac muscle mass gain have propelled technological and software developments aimed at understanding protein turnover, which can be applied to other fields. There have been particular developments in isotope labeling and kinetic modeling methods, which have elucidated the half-life of proteins in many various cellular compartments in the heart [74–77]. Developments in data analysis methods are particular important in this area, as although stable isotope administration and mass spectrometry approaches have been in use for decades, MS data measuring the rate of isotope incorporation cannot be efficiently analyzed on a very large scale without specialized software that can deconvolute isotope patterns and fit massive datasets to kinetic models [78, 79]. Following these advances, in vivo studies are revealing a previously unknown regulatory layer and architecture of the proteome in which functionally associated proteins share more synchronous turnover rates. During disease development, protein pathways have been found to deviate from physiological baseline via elevated protein replacement but not any apparent change in steady-state abundance, a result consistent with increased protein synthesis counterpoised by increased degradation [79]. Hence the measurement of half-life is also pursued to identify candidate disease proteins and from which to infer significantly dysregulated biological processes during pathogenesis.

### Mapping protein–protein interaction networks

There have been marked improvements in experimental protocols in affinity purification, as well as statistical and data science methods to filter out false positives in pulldown experiments. Chiang et al. recently elucidated the interactome of the protein phosphtase 1 catalytic subunit (PP1c), identifying 78 interacting partners in human heart. The proteomics results found increased binding to PDE5A in paroxysmal atrial fibrillation patients to impair proteins involved in electrical and calcium remodeling, a result that has implications in the understanding and treatment of atrial fibrillation [80]. Waldron et al. identified the TBX5 interactome in the developing heart to discover its interactions with the repressor complex NuRD, elucidating the mechanisms by which TBX5 mutations can influence cardiac development and confer congenital heart diseases. The accretion of public-domain protein–protein interactome data are also serving as a permanent resource that benefits other investigators outside the proteomics field, and in one but many recent examples provided important context to systems genetics experimental data to evidence the involvement of an interacting cilia protein network in congenital heart diseases [81]. More recently, the CoPIT method extends the scope of comparison to degrees of interactions among samples across cell states with more rigorous statistics, and is particularly notable in its suitability for quantifying differential interactomes of membrane proteins in human diseases [47]. Potential protein–protein interactions can now also be predicted in silico and de novo using machine learning algorithms that take in experimental data and auxiliary information [82].

At the same time, there is renewed interest to perform crosslinker studies on a large scale, which in addition to identifying protein–protein interaction partners, can provide information on the topology and protein domains involved in the interactions. Again we note that the development of new proteomics methodologies now necessitates hand-in-hand advances of novel data science solutions almost without exception. An example is the application of chemical cross-linkers in proteomics, which allows the linking of proximal proteins to quantify the degrees and likelihood of protein–protein interactions in their native cellular environment. Cross-linking proteomics experiments are however infeasible without specialized search engines that can consider the combinatorics of crosslinked peptide sequences, and identify interacting proteins whilst controlling for the FDR that result from the quadratic increase of search space [83, 84].

## Outlooks: emergence of proteomics big data

Quantitative shotgun proteomics has developed into a remarkably powerful technology that enables sophisticated questions on cellular physiology to be asked. The total volume of proteomics data generated per year now ranges in the petabytes. This is paralleled by an increasing number of available proteomics datasets in the public domain that can be reused and reanalyzed, with as many as 100 new datasets being made available per month on the proteomics data repository PRIDE [85]. Hence joining next-generation genomics, proteomics has become a veritable source of biomedical “big data”. As our capacity for data generation surges, opportunities for breakthroughs will increasingly come from not how much more data we can generate, but how well we can make sense of the results. As a corollary, the need for proteomics big data solutions is poised to skyrocket in the coming few years, where new resources, tools, and ways of doing science are needed to rethink how best to harness datasets and discern deeper meanings. The production of biological knowledge will involve tools and solutions devised in the field of data science, including those concerning data management, multivariate analysis, statistical learning, predictive modeling, software engineering, and crowdsourcing. Several current limitations and possible future frontiers, out of many, are discussed below:

Despite impressive gains, improvement of protein identification will likely continue to be an area of active research. It is estimated that up to 75–85 % of mass spectra generated in a proteomics experiment can remain unidentified by current data analysis workflows [62, 86], thus leaving room for continuous growth through better bioinformatics in the near future. Currently the unidentified “junk” spectra are mostly siloed or discarded, thus they constitute a major untapped source of biomedical big data. More inclusive search criteria (e.g., considering non-tryptic cleavage) can enhance identification, but there also exists a substantial portion of spectra that represent bona fide peptides not amenable to existing methods. These include peptides too short to score well in searching algorithms (≤5 amino acids), and peptides that are absent from protein sequence databases, e.g., variant peptides from polymorphisms or unknown splice isoforms. The advent of massive publicly available datasets has opened new avenues to tackle this problem [62, 63]. For instance, the millions of unidentified spectra that are uploaded to the PRIDE proteomics data repository may be systematically sorted and clustered, then analyzed via more exhaustive search protocols to identify what peptides are commonly present but unidentified across datasets and experiments.

Secondly, advances in protein quantification techniques, via both experimental and computational developments, will likely continue unabated. Many quantification techniques do not take into account the peptides that may become post-translationally modified or otherwise lost in a biological state. Currently, decreases in label-free measured quantity may be confounded by differences in protein modifications, digestion, or ionization, or matrix effects from different samples. For instance, the acquired spectral counts may be inflated by the existence of shared peptides among multiple (documented or undocumented) protein forms [87] as well as the sampling saturation for high-abundance peptides [88]. Statistical approaches pioneered in transcriptomics may be useful which can take into account the many-to-many mappings between proteins and peptides and to reconstruct proteoforms from individual peptide signals.

Lastly, the identification of unknown or unspecified PTMs will likely see continued progress. Because the multiplicity of the possible modification types on a peptide can shift peptide fragment masses combinatorially, they can greatly inflate the number of possible matches. Efforts are underway to develop custom sequence databases and devise new algorithms to extract information from existing cardiovascular proteomics datasets that is currently “hiding in plain sight”. For instance, algorithms can be used to predict peptide fragment intensity to improve peptide identification [89]. To make PTM search computationally tractable, multi-pass or cascade search approaches have been implemented that restrict the possibility of modified peptides to only within proteins that were preliminarily identified in the initial search. To improve peptide identification, “spectral libraries” including library modules for the cardiovascular system have been constructed that contain previously identified spectra, against which new experimental spectra can be directly matched for identification [57, 90, 91]. Because spectral libraries contain only a small subset of all theoretical sequences, and contain precise peptide fragment intensity in addition to ion masses, library search can lead to faster and more accurate identification.

To summarize, we recall an apt analogy provided by Loscalzo to compare the understanding of cardiac proteome with that of building a house and the genome with that of its floor plan [92]. Genomics kick-started the era of high-throughput omics investigations, but building a house requires more than just the blueprint; the complexity of protein regulation and pathway functions is better approached with proteomics. Technological advances in the last decade have gained tremendous power to discover finer minutiae of the house of the cardiac proteome. Success in the next 5 years will likely come from interfacing big proteomics data and computational approaches to distill regulatory principles and to support diagnostic/prognostic process from seemingly overwhelming information. The notion that existing data contain additional latent information that may be extracted to answer future questions is a fundamental tenet of big data science. Extrapolating from current developments, one can envision sophisticated discovery-driven studies in cardiac biomedicine, where original research projects may be initiated by data scientists using publicly-accessible proteomics datasets to ask new and unanticipated questions. A vibrant data science culture that promotes interactions between data generators and informaticians will facilitate the design and validation of computational methods and promote continued development in proteomics.

## Abbreviations

MS: mass spectrometry
LC: liquid chromatography
PTM: post-translational modification
MRM: multiple reaction monitoring
PDE: phosphodiesterase
cGMP: cyclic guanosine monophosphate
FDR: false discovery rates
2D: two-dimensional
SAF: spectral-abundance-factor
